# *Viburnum opulus* L. Juice Phenolic Compounds Influence Osteogenic Differentiation in Human Osteosarcoma Saos-2 Cells

**DOI:** 10.3390/ijms21144909

**Published:** 2020-07-11

**Authors:** Małgorzata Zakłos-Szyda, Adriana Nowak, Nina Pietrzyk, Anna Podsędek

**Affiliations:** 1 Institute of Molecular and Industrial Biotechnology, Department of Biotechnology and Food Sciences, Lodz University of Technology, Stefanowskiego 4/10, 90-924 Łódź, Poland; nina.pietrzyk@dokt.p.lodz.pl (N.P.); anna.podsedek@p.lodz.pl (A.P.); 2 Department of Environmental Biotechnology, Lodz University of Technology, Wólczańska 171/173, 90-924 Łódź, Poland; adriana.nowak@p.lodz.pl

**Keywords:** *Viburnum opulus*, phenolic compounds, Saos-2

## Abstract

Bone mass loss occurs with a decrease in osteoblast proliferation and differentiation, or the enhancement of bone resorption, which further leads to the impairment of bone mineral density and increase in bone fracture. Recent studies suggest that some phenolic compounds found in food play positive role in bone metabolism. High content of phenolic compounds with potential beneficial effects on bone metabolism have been identified in the *Viburnum opulus* fruit. The aim of the study was to determine the influence of *V. opulus* fresh juice (FJ) and juice purified by solid phase extraction (PJ) on osteogenesis processes with osteosarcoma Saos-2 cell lines. *V. opulus* purified juice revealed stronger potential as an inducer of Saos-2 osteogenic differentiation. Saos-2 cells matrix mineralization was evaluated with alkaline phosphatase (ALP) activity measurement and alizarin red S staining. Gene expression analysis showed the elevation of the mRNA levels of Runt-related transcription factor 2 (*RUNX2*), *ALP*, collagen type 1 and osteonectin, whereas the nuclear factor-κB ligand and osteoprotegerin ratio (*RANKL/OPG*) decreased. Furthermore, *V. opulus* was able to diminish the secretion of pro-inflammatory cytokines Il6 and TNFα, however had no effect on vascular endothelial growth factor (VEGF). It decreased intracellular oxidative stress and induced DNA repair, but had no effect on the growth inhibition of lactic acid beneficial microorganisms.

## 1. Introduction

Homeostasis between bone resorption and bone formation maintains bone tissue integrity and health, and is controlled both by physical stimulation and cellular signaling molecules [1,2]. The osteoclasts reducing the mineralized extracellular matrix and the osteoblasts producing organic bone are mainly involved in bone architecture modelling [3]. In osteoporosis, the reduction in osteogenesis occurs with an enhancement of bone resorption, leading to the impairment of bone mineral density and increase in bone fractures [4]. The major osteogenesis regulation factor is Runt-related transcription factor 2 (RUNX2), which regulates the proliferation of osteoblast progenitors and their differentiation into osteoblasts [5]. Differentiated osteoblasts are characterized by the increase in alkaline phosphatase (ALP) activity and increased expressions of bone markers, such as type I collagen (COL1A1), helping in mineral deposition, or osteonectin binding selectively to both hydroxyapatite and collagen 1, improving its mineralization [6]. At the cellular level, stimulation of osteoclasts is correlated with the upregulation of inflammatory cytokines, including interleukin 1 (Il1), interleukin 6 (Il6) and tumor necrosis factor α (TNFα) [7]. The other mechanisms leading to osteoclast activation and maturation involve signal transduction after the receptor activator of the nuclear factor-κB ligand (RANKL) binding with its RANK receptor, expressed by osteoclasts. The osteoprotegerin (OPG) secreted by osteoblasts captures RANKL, which limits its binding to the RANK receptor and, in turn, sustains bone mass. Due to the significant impact on morbidity, osteoporosis has been included in the most world-wide relevant nutrition-related chronic diseases, along with cancer, diabetes, obesity and cardiovascular diseases [4]. Whereas the etiology of osteoporosis is very complex, recent data link bone loss with the obesity state. Obesity-related enhanced adipocyte growth leads to the elevation of proinflammatory cytokine production, and thus intensifies osteoclast differentiation via RANKL/RANK/OPG signal transduction and, finally, bone resorption [8,9,10,11]. In the prevention and treatment of osteoporosis, there are used estrogens or selective estrogen receptor modulators; however, due to the side effects resulting from their prolonged usage, less harmful plant derived substitutes are being searched [12].

Epidemiological studies correlated a diet enriched in fruits and vegetables with a lowered risk of chronic disease development [13]. Its beneficial role for human health is mainly associated with the consumption of polyphenols [14,15,16,17,18,19]. These secondary plant metabolites’ protective role might be linked to antioxidant and anti-inflammatory properties; however, there is growing evidence about their direct influence on gene expression. Growing data reveal that phenolic compounds, especially tea catechins, procyanidins and chlorogenic acids, have a beneficial impact on restraining osteoporosis induced by oxidative-stress, as well as bone loss during a high-fat diet [1,20,21,22,23]. What is more, a recent study demonstrated that, in obese children treated with sweet cherry polyphenols, the osteoclastogenesis process was decreased, as well as the level of TNFα [10].

**Figure 1:**
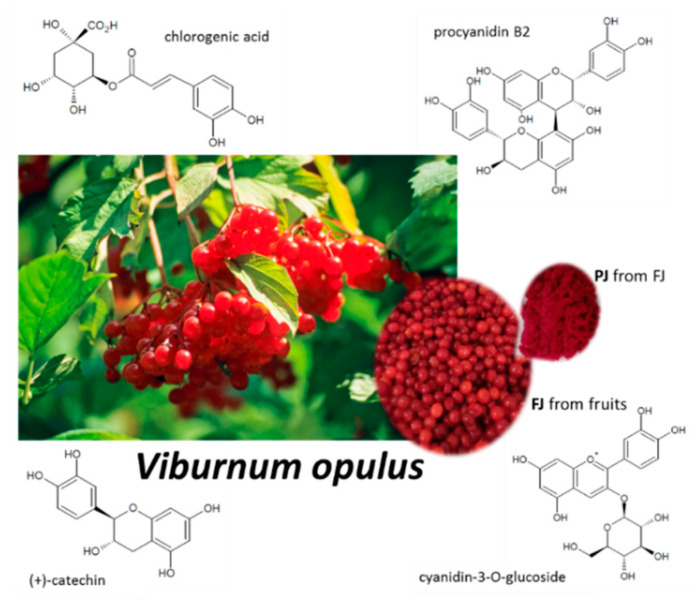
*Viburnum opulus* fruit as source of fresh juice (FJ) and purified juice (PJ); structures of the main phenolic compounds identified in *V. opulus* samples.

Our previous studies identified *Viburnum opulus* fruit as a rich source of phenolic compounds, with chlorogenic acid, proanthocyanidins and catechins as the main constituents, as shown in Figure 1 [24,25]. Despite their high antioxidant potential, *V. opulus* phenolics were able to decrease intracellular oxidative stress and inhibit cell migration [24,25,26]. Further studies revealed that *V. opulus* was involved in lipid and carbohydrate metabolism regulation in human epithelial Caco-2 and mice insulinoma MIN6 cells, where the alteration of selected gene expression was observed. Taking into account a significant impact of *V. opulus* phenolic compounds on the modulation of cellular metabolism, as well as its phenolic compounds composition, in the present study we investigated the effect of *V. opulus* fruit juice on the metabolic activity and mineralization process in human osteosarcoma Saos-2 cell lines. Saos-2 cells display osteoblastic features similar to primary human osteoblastic cells—they express active alkaline phosphatase and form the calcified matrix [27]. The presented studies assessed *V. opulus* juice’s influence on the activity of alkaline phosphatase and matrix mineralization, as well as the expression of genes related to osteogenic differentiation (*RUNX2, RANKL, OPG, osteonectin, ALP, TNFα, Il6*). Furthermore, the influence of *V. opulus* components on vascular endothelial growth factor (VEGF) secretion by human umbilical vein endothelial cells (HUVEC) was elucidated.

The symbiosis between gut microbiota and the host requires a homeostasis, which, once disrupted, may increase the risk of osteoporosis. The imbalance in the human gut microbiota can lead to the release of active compounds that contribute to multiple organ dysfunctions. Furthermore, in view of the fact that gut microbiota can play a significant role in osteoporosis, the antimicrobial activity of *V. opulus* juice phenolics towards selected pathogens, contributing to inflammation in the human body, was estimated. Additionally, the inhibitory effect of *V. opulus* on beneficial *Lactobacillus* sp. strains was evaluated

*V. opulus* fruits are used in food products, such as herbal tea, juice, cordials and liqueurs, fermented drinks, jams, marmalades and sauces [28]. However, due to the fruit’s bitterness and astringent properties, the obtained fresh juice has a very specific taste and needs to be diluted before consumption. Therefore, the effective concentration of *V. opulus* phenolics can be difficult to achieve in the gut. What is more, a much lower concentration of phenolic compounds present in fresh juice would require the consumption of increased amounts of juice. Solid phase extraction turned out to be a very effective method of increasing the concentration of phenolic compounds in the final formulation. The resulting purified juice (PJ) preparation, obtained from fresh juice (FJ), can be a component of functional food for people with osteoporosis. That preparation can also be used to check the direct interaction of the *V. opulus* phenolic components with other isoflavones, which are present in many pharmaceuticals intended for menopausal women. Thus, in the present study we investigated *V. opulus* fruit fresh juice (FJ) and purified juice (PJ) effects on the Saos-2 cells mineralization process. 

## 2. Results

### 2.1. Content of Phenolic Compounds in Fresh and Purified Juices of Viburnum opulus Fruit

Individual phenolic compounds’ contents are presented in Table 1. Their identification, based on retention times, wavelengths of maximum absorbance, deprotonated molecules ([M-H]^−^) and diagnostic fragments (MS/MS) was described in our previous paper [29]. The total phenolic contents were 11.508 ± 0.154 mg/g freeze-dried fresh juice (FJ) and 878.632 ± 2.722 mg/g freeze-dried purified juice (PJ). The FJ and PJ showed the presence of different groups of phenolic compounds, such as hydroxycinnamic acids, flavanols, flavonols- and anthocyanins. The mentioned groups of phenolic compounds participated with 77.50, 19.52, 0.37 and 2.61% of the total phenolic in FJ, and with 80.46, 16.30, 0.32 and 2.92% in PJ, respectively. Significant differences (*p* ≤ 0.05) in the contents of individual phenolic compounds between FJ and PJ were noted as a result of about a 90-fold increase in the content of phenolics in PJ. Chlorogenic acid showed the highest content, both among phenolic compounds and hydroxycinnamic acids. Its concentrations were 645.492 ± 1.984 mg/g and 8.039 ± 0.145 mg/g in PJ and FJ, respectively. Procyanidin dimer B1 and (+)-catechin were quantitatively dominant polyphenols in the flavanol group. In the tested FJ and PJ, cyanidin-3-glucoside and quercetin-3-vicianoside were the main anthocyanin and flavanol, respectively.

### 2.2. V. opulus Influence on Cellular Metabolic Activity and Proliferation

To understand the impact of *V. opulus* on Saos-2 cells we first compared the influence of fresh juice and purified juice on mitochondrial enzyme activity with Presto Blue reagent.

**Figure 2:**
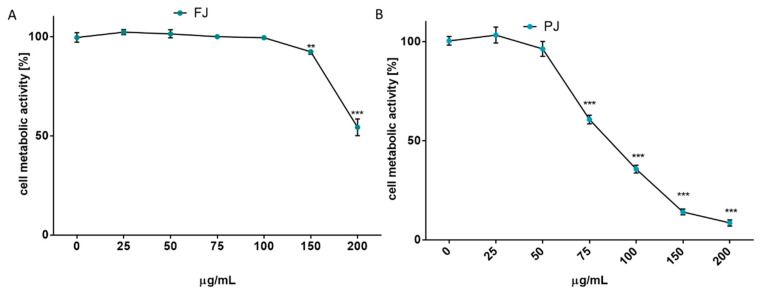
The influence of *V. opulus* on Saos-2 cell metabolic activity, determined by Presto Blue reagent after 48 h exposure with FJ (**A**) and PJ (**B**); control cells were not exposed to any compound; values are means ± SEM, *n* ≥ 12; statistical significance was calculated versus control cells (untreated), ** *p* ≤ 0.01, *** *p* ≤ 0.001.

After reaching confluence, the cells were incubated for 48 h with *V. opulus* samples increasing in concentration from 10 to 200 μg/mL. The results in Figure 2A show that at the highest dosage FJ decreased metabolic activity by almost 45%. No cytotoxic effect on Saos-2 cells was observed for the FJ concentration up to 100 µg/mL. At the same time, the PJ revealed higher cytotoxic potential than fresh juice, with the highest non-cytotoxic concentration equal to 50 µg/mL, as shown in Figure 2B. Cell incubation with 200 µg/mL PJ revealed the cell metabolic activity decreased by almost 90%.

**Figure 3:**
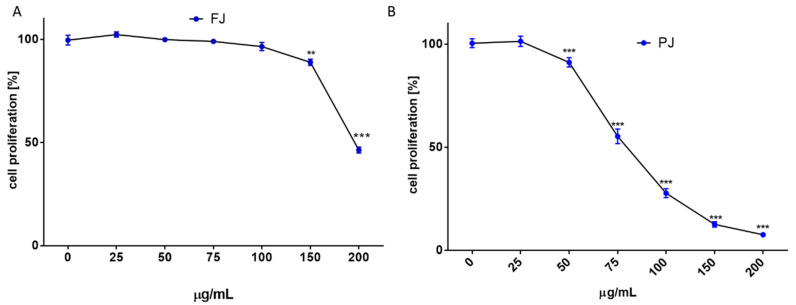

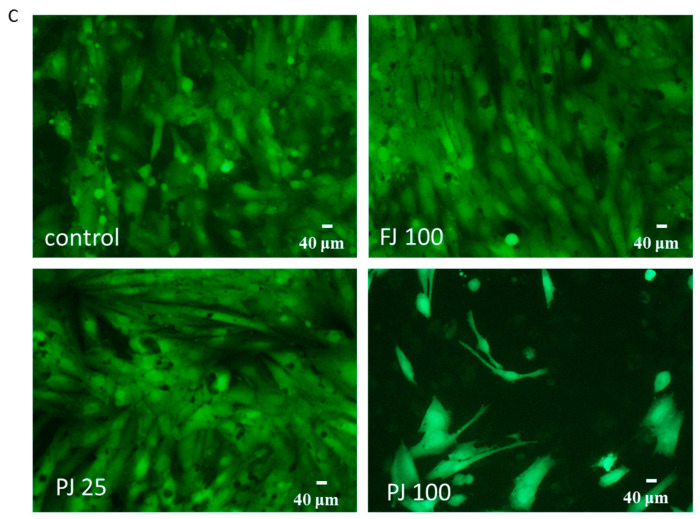
The influence of *V. opulus* on Saos-2 cell proliferation, determined by CyQuant reagent after 48 h exposure with FJ (**A**) and PJ (**B**); control cells were not exposed to any compound; values are means ± SEM, *n* ≥ 12; statistical significance was calculated versus control cells (untreated), ** *p* ≤ 0.01, *** *p* ≤ 0.001. Morphology of Saos-2 cells stained with 2 μM calcein AM observed after incubation with 25 and 100 μg/mL of PJ, and 100 μg/mL of FJ (**C**); randomly chosen fields were photographed at ×400 with fluorescent microscope.

Due to the long incubation of cells with *V. opulus* samples, their influence on cell proliferation was additionally checked with CyQuant Proliferation Assay, which relies on the fluorescence measurement of the cyanine dye bound to cellular nucleic acids. The *V. opulus* samples in the tested concentration range had no stimulatory effect on the proliferation of Saos-2 cells. As is presented in Figure 3A,B, purified juice negatively affected the DNA content at 50 µg/mL doze, decreasing the cell proliferation by 15%, whereas the comparable result of FJ was observed for 150 µg/mL concentration. A comparison of *V. opulus* inhibitory effects on cell proliferation and metabolic activity demonstrated that both samples decreased cell division more efficiently than metabolism, as shown in Figure 2 and Figure 3. Thus, taking into account the obtained results, the highest non-cytotoxic concentrations (IC_0_) chosen for the studies of osteoblast activity regulation were 100 μg/mL of fresh juice and 25 μg/mL of purified juice.

Microscopic observations performed with calcein AM ester confirmed the lack of cytotoxic effects of *V. opulus* samples at IC0 concentration on Saos-2 cells, as shown in Figure 3C. In healthy cells with active esterases, there is a visible strong cytosolic green-fluorescence of calcein. Cells incubated with 100 µg/mL of PJ preparation had lower cytoplasmic esterase activity, thus the decreased green-fluorescence of calcein was observed, as well as a lower number of attached cells. In that case, the PJ tested dose inhibited the cell proliferation to 25% and reduced the metabolic activity by 60%.

### 2.3. V. opulus Influence on Alkaline Phosphatase Activity

The effect of *V. opulus* on the activity of alkaline phosphatase, which is essential for osteoblast mineralization, was tested with p-nitrophenyl phosphate (pNPP) assay.

**Figure 4:**
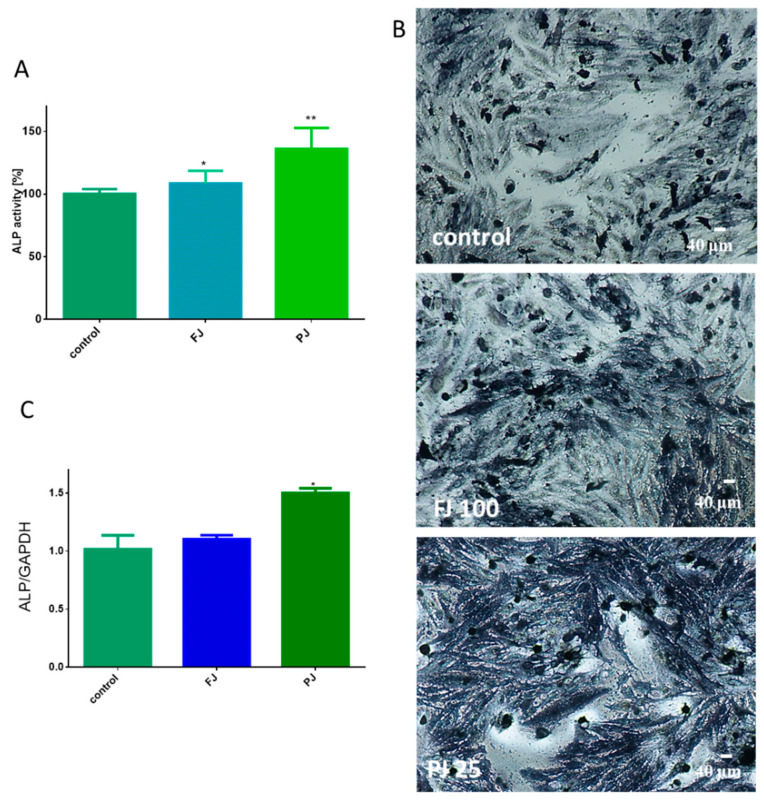
The influence of *V. opulus* FJ and PJ at IC_0_ dosage on Saos-2 cell alkaline phosphatase (ALP) activity, determined with pNPP assay after 8 days of exposure (**A**); control cells were not exposed to any compound; values are means ± SEM, *n* ≥ 12. Cells observed in the presence of BCIP/NBT, known substrate for ALP, after their incubation with 100 μg/mL FJ and 25 μg/mL PJ (**B**); randomly chosen fields were photographed at ×400 with contrast-phase microscope. The expression of mRNA level of *ALP* gene quantified by real-time PCR and normalized using glyceraldehyde-3-phosphate dehydrogenase (*GAPDH*) as a reference gene (*n* = 4) (**C**). Statistical significance was calculated versus control cells (untreated), * *p* ≥ 0.05, ** *p* ≥ *0.01.*

As it is shown in Figure 4A, purified juice at IC_0_ dosage significantly increased ALP activity by 35%, whereas fresh juice was able to elevate enzyme activity by almost 8%. Cell incubation with ALP substrate (BCIP/NBT) and imaging with bright-field microscopy also confirmed the promotion of ALP activity by the samples. It can be seen in Figure 4B that cells treated with purified juice generated the largest amount of dark blue stained ALP product. Further studies showed that the observed elevation of enzyme activity was correlated with an *ALP* gene expression increase at the transcription level, as shown in Figure 4C. Cells treated with the *V. opulus* purified juice exhibited a 1.5-fold increase in *ALP* mRNA expression, compared to the control cells. Fresh juice seemed to have no significant influence on the *ALP* mRNA expression level.

### 2.4. V. opulus Influence on Matrix Mineralization

The ability of Saos-2 cells to produce calcified extracellular matrix was confirmed with alizarin red S staining, which allows for the measurement of the amount of calcium deposited in the extracellular matrix.

**Figure 5:**
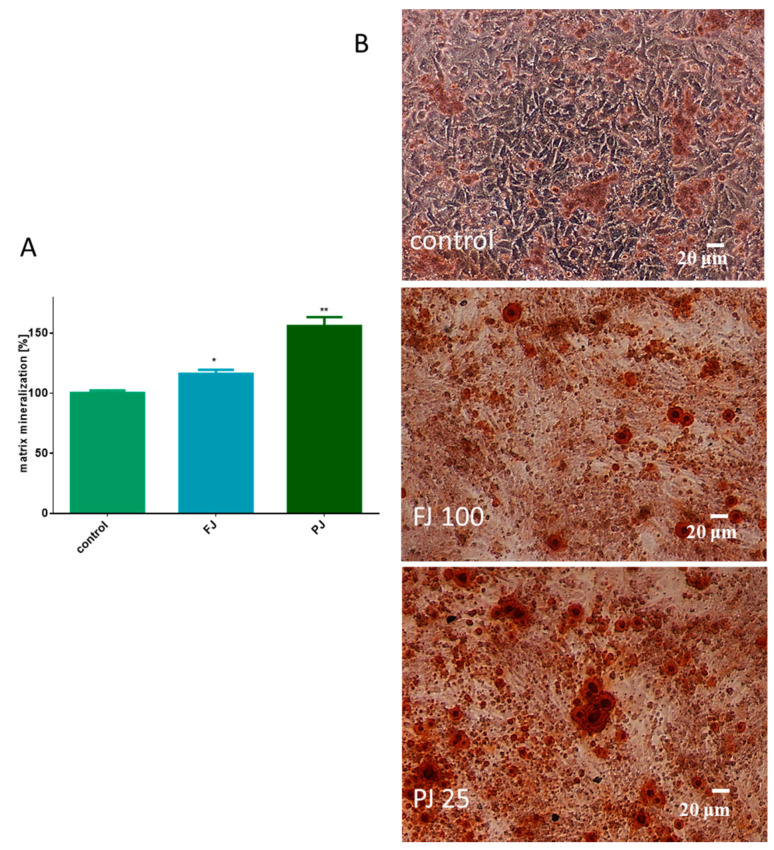
The influence of *V. opulus* FJ and PJ at IC_0_ dosage on Saos-2 cell matrix mineralization, determined by alizarin red S assay after 8 days of exposure (**A**); control cells were not exposed to any compound; values are means ± SEM, *n* ≥ 12; statistical significance was calculated versus control cells (untreated), * *p* ≥ 0.05, ** *p* ≥ 0.01. Cells stained with alizarin red S after their incubation with 100 μg/mL FJ and 25 μg/mL PJ (**B**); randomly chosen fields were photographed at ×200 with contrast-phase microscope.

Both *V. opulus* samples were able to stimulate the mineralization process after 8 days of treatment of Saos-2 cells. As presented in Figure 5A, fresh juice elevated the matrix mineralization by almost 15% in comparison to the control cells. That quantification data were followed by microscopic observations, which confirmed the appearance of red-stained round-shape granules related to the bone nodule formation, as shown in Figure 5B. After treatment with purified juice, Saos-2 cells appeared to have a 60% increase in matrix calcification compared to the untreated cells. Purified juice preparation increased the size and number of mineralized granules, demonstrating its ability to stimulate osteogenic differentiation.

### 2.5. V. opulus Influence on Expression of Genes Associated with Osteogenesis

**Figure 6:**
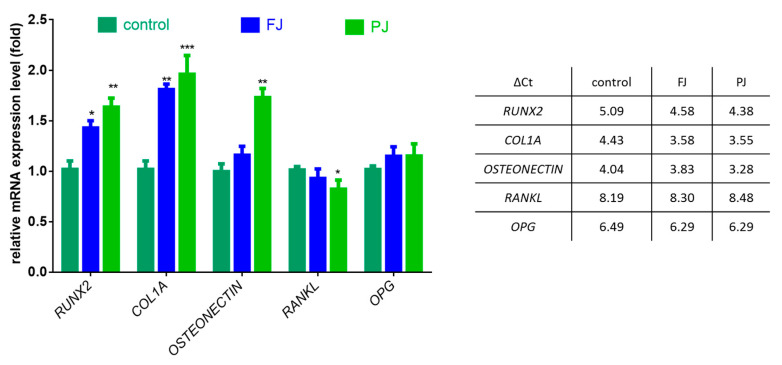
The influence of *V. opulus* FJ and PJ at IC_0_ dosage on the expression of selected genes in Saos-2 cells after 8 days of exposure, quantified with real-time PCR and normalized using glyceraldehyde-3-phosphate dehydrogenase (*GAPDH*) as a reference gene; ΔCt values are presented in table. Control cells were not exposed to any compound; values are means ± SEM, *n* = 4; statistical significance was calculated versus control cells (untreated), * *p* ≤ 0.05, ** *p* ≥ 0.01, *** *p* ≥ 0.001

After cell treatment with *V. opulus* samples, the mRNA expression of selected osteogenic marker genes was determined by real-time PCR—Runt-related transcription factor 2 (*RUNX2*), type 1 collagen (*COL1A1*), osteonectin, receptor activator of nuclear factor kappa-Β ligand (*RANKL*) and osteoprotegerin (*OPG*). The data show that purified juice had a greater effect on gene expression at the transcription level than fresh juice, as shown in Figure 6.

The FJ sample increased the expression of *RUNX2* mRNA levels by 40%, whereas PJ by almost 60%. Both samples upregulated the expression of *COL1A1* mRNA by 80–95%. The mRNA level of osteonectin was influenced only by PJ, which amplified the expression by 75%. *V. opulus* samples had no effect on the expression of *OPG*, whereas a 15% decrease in the *RANKL* mRNA level was observed only for purified juice.

### 2.6. V. opulus Influence on Intracellular Reactive Oxygen Species Production and DNA Repair

**Figure 7:**
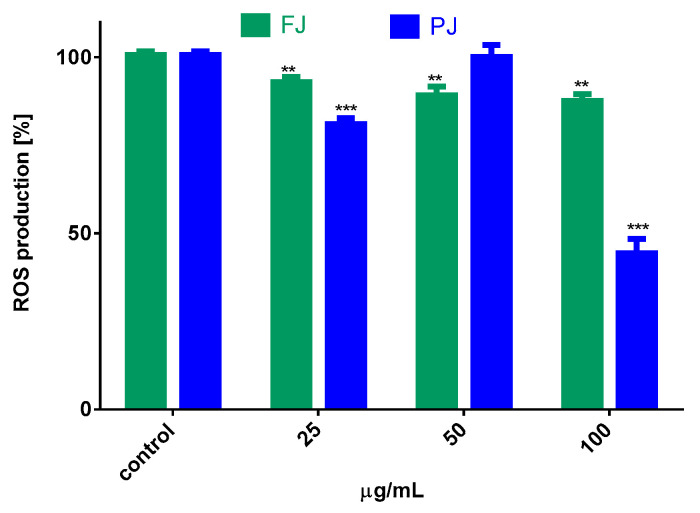
The influence of *V. opulus* FJ and PJ at IC_0_ dosage on intracellular ROS generation in Saos-2 cells after 8 days of exposure, quantified with DCFH-DA assay; control cells were not exposed to any compound; values are means ± SEM, *n* ≥ 9; statistical significance was calculated versus control cells (untreated), ** *p* ≤ 0.01, *** *p* ≤ 0.001.

The influence of *V. opulus* juice on the regulation of intracellular reactive oxygen species (ROS) production by Saos-2 cells was determined with dichloro-dihydro-fluorescein diacetate (DCFH-DA) assay. The results in Figure 7 show that cells treated with both samples of *V. opulus* at IC_0_ dosage declined intracellular ROS level by 10–20% in comparison to the control cells. The purified juice was more efficient as an oxidative stress reducer than fresh juice. Additionally, data show that the level of ROS changed depending on the PJ concentration studied. Cell treatment with 50 µg/mL PJ, a dose with previously demonstrated low cytotoxicity and decreasing metabolic activity of cells, as shown in Figure 2 and Figure 3, elevated intracellular ROS generation, due to the induced mitochondria dysfunction. The further elevation of PJ dosage (100 µg/mL) significantly decreased the number of living cells, as well as attached cells, and thus the ROS level. Therefore, the observed significant reduction in intracellular ROS is not related to antioxidant ability but confirmed PJ cytotoxic potential. In our previous studies on *V. opulus* fruit preparation activity, we observed comparable patterns of their influence—low doses of preparations were cytoprotective against cells, whereas increased triggered mitochondrial membrane depolarization decreased the ATP level and activated caspase 9 and, finally, apoptosis [30].

**Figure 8:**
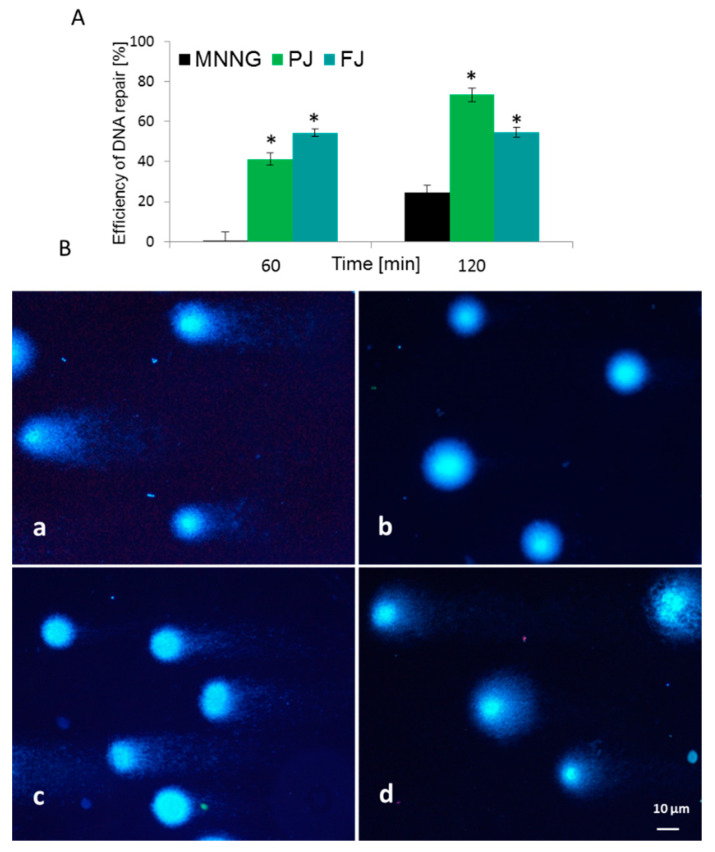
Efficiency of DNA repair (%) in Saos-2 cells exposed to MNNG mutagen and post-incubated with *V. opulus* FJ and PJ at IC_0_ concentration (**A**). Typical images of comets stained with DAPI (**B**): (**a**) 6.8 µM MNNG; (**b**) negative control on untreated cells; (**c**) PJ influence after 60 min and (**d**) 120 min of post-treatment. Fluorescence microscopy (Nikon, Tokyo, Japan); ×200 magnification. * *p* ≤ 0.01.

To evaluate *V. opulus* cytoprotective potential against cellular DNA damage, firstly Saos-2 cells were challenged with methylnitronitrosoguanidine (MNNG) mutagen, then post-incubated with FJ and PJ samples at IC_0_ dose. DNA repair was measured at time zero, after 60 and 120 min of incubation. For the MNNG positive control, approximately 25% efficiency of DNA repair was observed after 120 min in comparison to the initial point. As presented in Figure 8A, both samples induced DNA repair in a statistically significant manner. *V. opulus* induced DNA repairs very efficiently after 60 min of incubation, and it was from 40 to 55% for PJ and FJ, respectively. The effect was significantly enhanced over 120 min, where, for cells incubated with purified juice, DNA damage was approximately 50% lower than for the positive control. At that time point, the PJ sample was the strongest DNA repair inducer. Example images of typical comets are presented in Figure 8B.

### 2.7. V. opulus Influence on Pro-Inflammatory Markers: Il6, TNFα and VEGF Secretion

**Figure 9:**
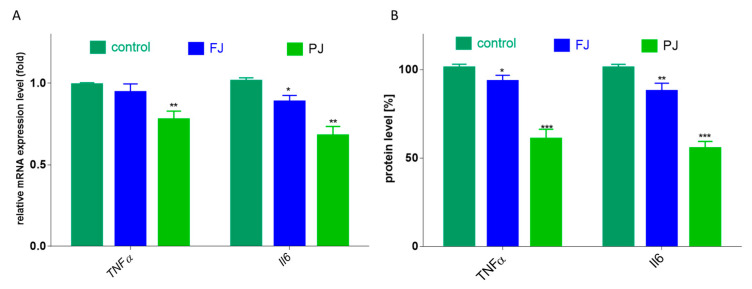
The influence *V. opulus* FJ and PJ at IC_0_ on mRNA expression, quantified with real-time PCR and normalized using glyceraldehyde-3-phosphate dehydrogenase (*GAPDH*) as a reference gene (**A**) and protein secretion quantified with ELISA (**B**) of TNFα and Il6 in Saos-2 cells after 8 days of exposure; control cells were not exposed to any compound; values are means ± SEM, *n* = 4; statistical significance was calculated versus control cells * *p* ≤ 0.05, ** *p* ≤ 0.01, *** *p* ≤ 0.001.

Among cytokines, the most related to bone health are tumor necrosis factor α (TNFα) and interleukin 6 (Il6). In the case of *TNFα*, only purified juice significantly declined the mRNA expression level by 25, compared to the control cells, as shown in Figure 9A. Taking into account the influence of *V. opulus* on protein secretion by cells, the 40% reduction in TNFα release was induced by PJ, whereas FJ downregulated this cytokine level to 90%, as shown in Figure 9B. Simultaneously, both *V. opulus* probes diminished *Il6* gene expression (by 15–30%), as shown in Figure 9A, which was accompanied by a reduction in Il6 protein secretion by Saos-2 cells to 55–80%, as shown in Figure 9B. As before, purified juice was a more effective inhibitor of interleukin 6 secretion by cells than FJ.

**Figure 10:**
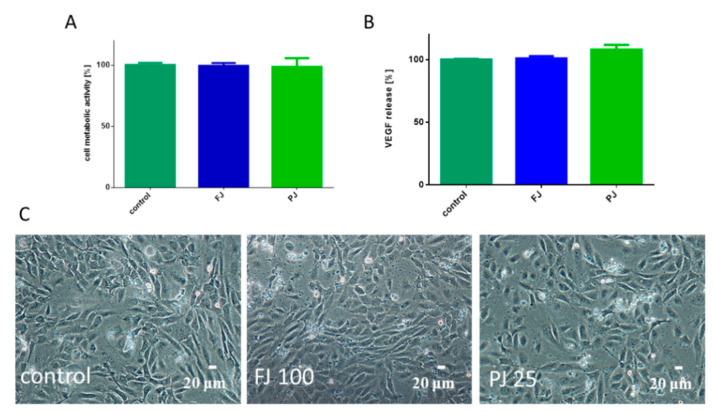
The influence *V. opulus* FJ and PJ at IC_0_ concentration on cellular metabolic activity, determined with Presto Blue assay (**A**) and VEGF secretion quantified with ELISA (**B**) by HUVEC cells; control cells were not exposed to any compound; values are means ± SEM, *n* ≥ 3. Morphology of HUVEC cells treated with *V. opulus* for 48 h (**C**); randomly chosen fields were photographed at ×200 magnification.

Because bone formation via osteogenesis is sustained by angiogenesis, thus the influence of *V. opulus* on human umbilical vein endothelial cells (HUVEC) was studied. Both *V. opulus* samples at IC_0_ concentration, determined previously for Saos-2 cells, had no effect on HUVEC metabolic activity, as shown in Figure 10A, as well as cell morphology, as shown in Figure 10C. *V. opulus* phytocompounds had no influence on the secretion of the vascular endothelial growth factor (VEGF) involved in angiogenesis, as shown in Figure 10B.

### 2.8. Antimicrobial Activity of V. opulus Against Lactic Acid Bacteria and Pathogens

Selected in our study beneficial bacteria (lactic acid bacteria) are a part of human gut microbiota, while the pathogens can contribute to dysbiosis, inflammation and acute diarrhea. Harmful bacteria can be responsible for the imbalance in the microbiota and can lead to the production of diverse metabolites causing dysbiosis and inflammation. The inflammation state (especially chronic) can be one of the main causes for this in the organism, which causes loss in bone biomass. Thus, in next step, we wanted to check the inhibitory activity of *V. opulus* juice in relation to beneficial and pathogenic bacteria. The antimicrobial activity of *V. opulus* is presented in Table 2. The obtained results indicate that, within pathogenic bacteria, Gram-positive were more sensitive to *V. opulus*, than Gram-negative bacteria. *S. aureus* ATTC 6538 was the most sensitive to both samples with average inhibition zones of 12.5 and 9.0 mm for FJ and PJ, respectively. *Ent. faecalis* ATCC 29212 was the most sensitive to PJ, with the mean inhibition zone 10.0 mm. *L. monocytogenes* ATCC 19115 was sensitive only to purified juice. The remaining pathogenic strains (i.e., Gram-negative as well as *C. albicans* ATCC 10231 yeast), were completely resistant to both tested samples. Generally, within pathogenic bacteria, *V. opulus* effectively only inhibited the growth of Gram-positive microorganisms. 

Gram-positive lactic acid bacteria (LAB), which are the inhabitants of the human gastrointestinal tract and are considered to be beneficial for health, were totally resistant to tested *V. opulus* samples.

## 3. Discussion

There is growing evidence of some phenolic compounds’ beneficial impact in the prevention of oxidative stress-induced bone loss and osteoporosis [1,2,31]. Among fruits rich in these secondary metabolites and present in the human diet is *V. opulus* [24,32], thus the primary aim of the study was to evaluate its effect on the activity of human osteoblastic type Saos-2 cell lines. The *V. opulus* influence on Saos-2 mineralization was tested with two samples: fresh juice and purified juice (via solid-phase extraction). Nearly 30 phenolic compounds in *Viburnum opulus* fruit were identified, with the presence of chlorogenic acid, procyanidins, catechins and cyanidin glycosides being the most prominent [24,25]. As presented, the phenolic rich sample (PJ) obtained from FJ revealed stronger influence on the metabolic activity and proliferation of Saos-2 cells. Whereas none of the samples induced cellular proliferation, they caused a concentration-dependent influence on cellular activity, according to the content of phenolic compounds. Probably the most responsible for the observed activity of *V. opulus* is chlorogenic acid—quantitatively the main component of FJ and PJ—as well as procyanidins and catechins. Despite the fact that the concentration of phenolic compounds in PJ was almost 90-fold higher than in fresh juice, PJ dosage, able to decrease cellular activity by 50%, was only 2.5-times lower than FJ. Similarly, the obtained IC_0_ value for fresh juice was only 4-times higher than for PJ. Thus, it appears that the observed biological impact of *V. opulus* fresh juice is related to the presence of other non-phenolic compounds, such as sugars, proteins, organic acids, minerals, or other phenolics lost during solid-phase extraction and not detected in PJ. Specifically, potential synergic activities and chemical interactions may be responsible for the observed cellular effect. Still, the presented results are in agreement with our previous studies, where a phenolic rich fraction obtained from *V. opulus* was more active in Caco-2 and MIN-6 cells [24,30]. 

Saos-2 cells can be differentiated to osteoblast-type cells in the presence of ascorbic acid and β-glycerol-phosphate [33]. Because the membrane-bound alkaline phosphatase enzyme is known as an early marker of osteoblast differentiation, essential for matrix mineralization, in the next step *V. opulus* influence on its activity was checked. As it was verified by ALP staining and quantitative activity analysis, both samples increased enzyme activity, leading to the elevation of the cellular phosphate concentration required for the initiation of hydroxyapatite formation. Indeed, alizarin red S staining confirmed the enhanced generation of the calcified extracellular matrix, resulting from the accumulation of mineralizing ions, such as Ca^2+^, HCO_3_^−^, CO_3_^2−^ or PO_4_^3−^ [34]. Meanwhile, *V. opulus* induced the expression of mRNA level of osteogenic genes, such as *RUNX2*, *COL1A1* and osteonectin, which indicated the promotion of Saos-2 cell osteoblast differentiation. Between the abovementioned proteins, the Runt-related transcription factor 2 is known as a key osteoblast differentiation regulator [18]. It binds to osteoblast specific cis-acting element (OSE) in the promoter region of the major osteoblast bone matrix protein genes and controls their expression [5]. The performed examination confirmed the enhancement of the *RUNX2* level in Saos-2 cells incubated with FJ and PJ preparations. The treatment of Saos-2 cells with *V. opulus* samples was able to elevate *ALP* gene expression. Simultaneously, further analysis established that collagen I gene expression on the transcription level was also upregulated. It is known that the production of type 1 collagen, which compromises 90% of the bone matrix, is the most important function of osteoblasts. Moreover, among other proteins regulated by RUNX2 transcription factor are osteonectin, promoting collagen mineralization, osteopontin inhibiting hydroxyapatite crystal growth and osteocalcin stimulating bone mineral maturation [18]. Purified juice was able to elevate the expression of the osteonectin mRNA level. Overall, *V. opulus* juice phytocompounds could be considered as agents of accelerating the mineralization process, enhancing osteoblast maturation through *RUNX2* increase and, finally, of the suspected elevation of RUNX2 protein expression. We are aware that the main limitation of the presented study is the attempt to explain the *V. opulus* molecular mechanism associated with the stimulation of the Saos-2 osteogenesis process basing mainly on the selected genes transcription level analysis. Given that the presented results contribute to elucidating *V. opulus* as an inducer of bone matrix mineralization, further evaluation on protein levels should be performed. What is more, in the observed influence on the mineralization process, other mechanisms can be triggered, such as increased signaling of bone morphogenetic proteins (BMP), which results in the upregulation of *RUNX2* factor. It was demonstrated that cryptochlorogenic and neochlorogenic acids present in dried plum extract upregulated MC3T3-E1 osteoblast activity by enhancing membrane-bound BMP2 signaling leading via phosphorylation to induce the expression of RUNX2 [35].

To the best of our knowledge, there are no reports of *V. opulus* fruit influence on osteogenesis regulation. Because the composition of FJ and PJ is very complex (more than 30 phenolic compounds were identified), it would be premature to infer that the osteogenic potential reported here results only from the main components present in preparations. However, there are data demonstrating the pro-osteogenic ability of some of the phenolic constituents, which were identified in *V. opulus* fruit. It is known that chlorogenic acid promoted osteogenesis in human adipose tissue-derived mesenchymal stem cells (hAMSCs), which was followed by the increase in mineralization with *ALP* and *RUNX2* upregulation [36]. Studies performed with a melon extract containing chlorogenic acid demonstrated its protective activity against bone loss in rats with induced osteoporosis [37]. After the treatment of animals, calcium concentration and strength of bone were elevated, but ALP blood level was decreased. Rats with induced osteoporosis treated with chlorogenic acid (9−45 mg/kg/d) had partially improved bone remodeling with ALP and osteocalcin elevation [38]. Studies performed in vitro with BMS cells demonstrated that chlorogenic acid induced cell differentiation by activating the cyclin D1 and phosphoinositide 3-kinase (PI3K)/Akt pathways [38]. Green tea catechin, (−)-epicatechin gallate (ECG), was shown to stimulate the osteoblast differentiation of C3H10T1/2 and hMSC cells with the upregulation of RUNX2 transcription factor and the activation of p38 MAP kinase, which is critical for osteoblast differentiation [39]. In the observed mechanism, the activation of the transcription coactivator with PDZ-binding motif (TAZ) was involved. The TAZ protein interacts with the RUNX2 protein, but also acts with the peroxisome proliferator activated receptor γ (PPARγ)—it promotes osteogenesis through RUNX2-mediated gene transcription and suppresses adipogenesis through the PPARγ nuclear receptor [40,41]. Another catechin, (−)-epigallocatechin-3-gallate (EGCG), increased the osteogenic differentiation of bone marrow hMSC cells leading to matrix mineralization through the upregulation of *RUNX2*, *ALP*, osteonectin and osteocalcin mRNA levels [42]. Additionally, the expression of promoting osteoblast-maturation bone morphogenetic protein 2 (*BMP2*) at mRNA level was enhanced.

Bone formation by osteoblasts can be enhanced by the inhibition of bone resorption controlled by osteoclasts [43]. After the binding of the receptor activator of nuclear factor-kB ligand (RUNKL), present on the surface of an osteoblast, to the RANK receptor, found in osteoclasts, the activation of osteoclastogenesis occurs [44]. Osteoclast differentiation can be inhibited by osteoprotegerin, which captures and binds RANKL, preventing its binding with the receptor present in osteoclast. As it was demonstrated, the PJ sample decreased the *RANKL* mRNA level, while not showing any significant effect on *OPG.* The compound regulation of osteoclastogenesis is connected with its influence on the RANKL:OPG ratio [45]. According to these calculations of mRNA expression levels, the *RANKL:OPG* ratio after cell treatment with PJ was reduced by almost 30% in comparison to the control cells. Hence, it can be assumed that *V. opulus* components may stimulate bone formation via the stimulation of mineralization. Its anti-resorptive function—thus osteoclastogenesis inhibition preventing bone loss—needs to be verified with osteoclast cell line studies. Whereas we did not perform experiments in this regard, the chlorogenic acid potential in the reduction in osteoclast differentiation was demonstrated [46]. In the molecular mechanism of bone marrow macrophages (BMMs), osteoclast differentiation inhibition involved the suppression of RANKL-mediated NF-κB activation. Further studies performed in mice demonstrated that chlorogenic acid administrated orally at a dosage of 10 mg/kg was able to suppress lipopolysaccharide-mediated bone erosion [36]. The osteoclastogenesis process inhibition by EGCG was identified, with decrease of the RANKL/OPG ratio in murine stromal cells, ST2 cells and macrophage-like RAW 264.7 cells [45]. Grape pomace polyphenols were also shown to decrease RANKL/OPG ratio and elevate the expression of *BMP2* and *RUNX2* mRNA in human MSC cells [23].

Reactive oxygen species are known subcellular messengers, whose excessive production leads to ROS-induced DNA damage, protein and lipid peroxidation and cell death [18,47,48]. It was demonstrated that oxidative stress inhibits osteoblastic differentiation with NF-κB activation [1,49]. What is more, it activates osteoclastogenesis and strengthens bone resorption during osteoporosis, leading to bone structure collapse and the loss of bone mass [1,47]. Whereas the potential of *V. opulus* as the antioxidant and the scavenger of artificial free radicals was determined previously [24,25], here its positive influence on the intracellular oxidative stress of Saos-2 cells was verified. It may be suspected that *V. opulus* phenolics could directly react with intracellular free radical cations or could enhance the activity of the cellular detoxifying enzymes. As it was previously demonstrated, *V. opulus* treatment enhanced glutathione (GPx) peroxidase activity in insulinoma MIN6 cells [30]. Still, the observed decrease in intracellular ROS level was in accordance with detected *ALP* and *RUNX2* elevation and matrix mineralization [49]. These *V. opulus* features are relevant in regard to the prevention of bone damage induced by oxidative stress accompanying the elevation of blood glucose or free fatty acids levels during the obesity state [16]. Additionally, *V. opulus* revealed cytoprotective properties against DNA damage induced by MNNG. Increase in DNA repair process efficiency by *V. opulus* was noted previously in Caco-2 cells [24]. Cytoprotective properties against hydroperoxide-induced apoptosis in osteoblastic cells MC3T3-E1 revealed grape seed proanthocyanidins [50], the group of compounds also present in *V. opulus* juice. 

The elevation of proinflammatory cytokines Il6 and TNFα is considered an osteoresorptive factor [51]. These proteins stimulate osteoclastogenesis and enhance bone resorption with the elevation of RANKL expression in osteoblasts. The observed decrease in Il6 and TNFα proteins expression and secretion in Saos-2 cells treated with purified juice is reflected in the decrease in *RANKL* mRNA level. Polyphenolic extracts from black, green and rooibos tea, rich in catechins, induced mineralization and ALP activity in Saos2 cells, which was followed by TNFα and Il6 inflammatory cytokine reduction [52]. On the other hand, obesity is associated with low-grade chronic inflammation, where the enlargement of adipocytes induces the secretion of TNFα and Il6, leading not only to insulin resistance but also to bone loss [9,53]. The anti-inflammatory property of chlorogenic acid was connected with oxidative stress decrease, the attenuation of NF-κB activation and, finally, TNFα and Il6 production [54]. Thus, the downregulation of Il6 and TNFα secretion by *V. opulus* is relevant not only for bone metabolism but it also has an impact on obesity state, contributing to osteoporosis delay. In this regard, further studies are required to assess *V. opulus* influence on the adipogenesis process and cytokine production by adipocytes.

Bone regeneration is sustained by the formation of blood vessels serving as a source of nutrients, oxygen, signaling molecules and ions used for mineralization, as well as for bone forming precursor cells [55]. Therefore, we investigated the influence of *V. opulus* on HUVEC cells able to migrate and involved in blood vessel formation. *V. opulus* samples at IC_0_ dose effectively inducing Saos-2 differentiation had no influence on HUVEC cell metabolic activity, as well as on VEGF secretion. That endothelial cell-specific mitogen promotes angiogenesis and accelerates bone healing through the promotion of progenitor cell differentiation into osteoblasts [55]. Studies performed on HUVEC cells under hypoxic conditions identified chlorogenic acid as an inhibitor of the angiogenesis process, able to decrease VEGF secretion [56]. However, chlorogenic acid was not able to inhibit VEGF-dependent VEGFR-2 activation in HUVEC cells, triggering angiogenesis [57]. Taking into account the obtained results, it can be hypothesized that other juice components may stimulate VEGF secretion, influencing a final cellular response in this regard. Another factor relevant for angiogenesis is cellular migration. Studies performed with a blueberry extract rich in chlorogenic acid demonstrated the enhancement of HUVEC cell migration [58]. On the other hand, in a mouse model with osteoarthritis, the animals’ treatment with grapes procyanidins was shown to downregulate *VEGF, TNFα*, *Il6* and *RANKL* expression, protecting the cartilage integrity [59]. Thus, the observed in vitro efficacy of *V. opulus* phenolic compounds in bone mineralization needs further in vivo studies performed with animal models treated with a high-fat diet and with induced osteoporosis.

For studies on the osteogenesis process of Saos-2 cells, *V. opulus* samples at IC_0_ concentration (25 and 100 µg/mL) were chosen, thus without a cytotoxic impact on cell viability and division. Studies performed with another phenolic compound, biochanin A, showed that its consumption of 5 mg per day (which corresponds to 1 µM) prevented bone loss effectively at a safe range level of its intake [51]. Taking into account that the mean polyphenol intake of an adult subject is about 283–1000 mg total polyphenols/day, the *V. opulus* phenolic compound concentrations used in this work can be achieved in the gut under physiological conditions [14]. Given the human studies, the maximum plasma concentration (C_max_) of chlorogenic acids and metabolites varies depending on the dose, individual and analytical methodology used in the study, from nM to low µM levels [60]. Given the molar mass of chlorogenic acid (354.31 g/mol), the concentration ranges from 1 nM to 1 µM correspond to a concentration from 0.35 to 354 μg/L. According to Farah et al. [61] chlorogenic acids present in green coffee are strongly absorbed and metabolized in humans. Maximum plasma concentration (C_max_) varied significantly between subjects and phenolic acid structure. For example, the C_max_ of all chlorogenic acids ranged from 0.6 to 16.9 μM and, for dicaffeoylquinic acids, ranged from 0.3 to 22.8 μM. In this human study, the chlorogenic acid C_max_ (dominant phenolic compounds in our extracts) was 5.9 µM or 2.1 mg/L for a consumed dose of 42 mg chlorogenic acid. Thus, our effective dose of purified juice (25 mg/L) contained 16 mg/L chlorogenic acid. Additionally, *V. opulus* phenolics’ binding affinity to human serum albumin may effectively regulate their bioavailability and cellular metabolism [22,30]. Data indicate that phenolic compounds, as well as chlorogenic acids, are metabolized by gut microbiota, and then their aglycons and other metabolites are absorbed in the small intestine by passive diffusion or with transporters [62]. Subsequently, *V. opulus* compounds bound to blood albumin could be delivered to bone forming cells and regulate their metabolism. Despite observed compound biotransformation in the intestine, meanwhile, they influence the growth and viability of gut microbiota [63]. Therefore, next, the impact of *V. opulus* on selected microorganism growth was investigated. Among the studied pathogens the antimicrobial activity of both samples was detected against Gram-positive bacteria. It could be suspected that the observed antibacterial action involves phenolic compounds targeting the rigidity, permeability or integrity of bacterial cell wall and membrane, as well as interaction with enzymes involved in bacterial metabolism [64]. Chlorogenic acid and procyanidins from grape seeds were shown to act against *S. aureus* and *L. monocytogenes* [65,66]. Simultaneously, the growth of Gram-positive lactic acid bacteria was not inhibited. The intestinal presence of these bacteria and their metabolic byproducts defend the organism against freshly ingested pathogen microorganisms, as well as protects against intestinal injury and inflammation [67]. In this regard, the feature of *V. opulus* to inhibit pathogenic bacteria without the inhibition of beneficial microorganisms is very relevant. The presented results are in line with other studies performed on mice with a high-fat diet [68]. Animals treatment with chlorogenic acid (150 mg/kg/d) promoted an increase in the relative abundance of *Lactobacillaceae* and *Bacteroidaceae*, as well as body loss and the reduction in plasma lipid levels. What is more, microbial dysbiosis (infection) can lead to inflammation processes and contribute to bone loss. Additionally, alterations in composition and the diversity of microbiota is correlated with many inflammatory and metabolic disorders (e.g., inflammatory bowel disease, obesity), which can change the permeability of intestinal cells. The gut microbiota can interact with the host immune system and further influence the host health. It was proven that gut microbiota plays a crucial role in bone metabolism and mass through immune and endocrine systems and calcium balance (cellular transport and absorption), but the exact mechanism is still unclear [69]. Some beneficial bacteria, such as *Lactobacillus salivarius* UCC 118, stimulate calcium uptake by enterocytes in in vitro models [70], whereas *L. rhamnosus* GG was shown to stimulate bone improvement in estrogen-deficient mice [71]. What is more, some products of lactic acid bacteria fermentation were also able to increase intestinal calcium absorption [72]. There are many reports linked to the beneficial effect of probiotic bacteria on estrogen deficiency-induced bone loss, which was well summarized in the review article by Xu et al. [72]. Reduced bone mass density is rather associated with taxon-specific signatures in the gut microbiota [73]. The composition of the cell wall of many Gram-negative bacteria (lipopolysaccharide and endotoxin) play an important role in bone metabolism [74]. In our study, *V. opulus* samples did not inhibit the growth of beneficial *Lactobacillus* species, which means that, when consumed together, they fulfil their protective functions, also linked to osteoporosis. What is more, these *Lactobacillus* strains could probably be useful in the fermentation of *V. opulus* juice with new pro-health properties, which will be the subject of our further research. 

## 4. Materials and Methods

### 4.1. Chemicals and Reagents

All cell culture reagents were obtained from Life Technologies (Carlsbad, CA, USA). Methylnitronitrosoguanidine (MNNG), Low Melting Point (LMP) agarose, Normal Melting Point (NMP) agarose, NaCl, Triton X-100, EDTA, Tris, NaOH and 4’,6-diamidino-2-phenylindole (DAPI) were purchased from Sigma-Aldrich (St. Louis, MO, USA). Other chemicals used, if not stated otherwise, were obtained from Sigma-Aldrich (Steinheim, Germany).

### 4.2. Preparation of V. opulus Samples, Identification and Quantitative Determination of Individual Phenolic Compounds by UPLC–PDA-Q/TOF-MS

Fresh fruits of the *V. opulus* were collected from Rogów Arboretum, Warsaw University of Life Sciences (Rogów, Poland) and authenticated with the account number 18162. Fresh juice (FJ) was obtained from homogenized fruit pulp, which was further centrifuged (5000 rpm for 10 min). Solid phase extraction with C-18 Sep-Pak cartridge and methanolic elution (10 g capacity, Waters Corp., Milford, MA, USA; 12-Port Vacuum Manifold system) was performed to isolate phenolic compounds from juice, to lyophilize and to obtain purified juice (PJ). The identification of individual phenolic compounds was performed according to the protocol described in our previous work [29] using a Waters UPLC^TM^ system (Waters Corporation, Miliford, MA, USA) with an Acquity UPLC HSS T3 C18 column (150 × 2.1 mm, 1.8 µm; Waters Corp., Milford, MA, USA) and a quadruple-time-of-flight mass spectrometer (Q/TOF-MS) (Waters Corp., Milford, MA, USA), equipped with electrospray ionization (ESI). The mobile phase consisted of (A) 0.1% formic acid in water and (B) acetonitrile (B). The UPLC elution conditions were as follows: initial conditions 99% (A); 12 min 65% (A); 12.5 min 100% (B); 13.5 min 99% (A). The flow rate was 0.45 mL/min. The mass spectrometer was operating in the negative mode for a mass range of 150–1500 Da, fixed source temperature at 100 °C, desolvation temperature at 250 °C, desolvation gas flow of 600 L/h, cone voltage of 45 V, capillary voltage of 2.0 kV and a collision energy of 50 V. Leucine enkephalin was used as a lock mass. The instrument was controlled by Mass-LynxTM V 4.1 software (Waters Corp., Milford, MA, USA). Phenolic compounds were monitored for flavanols at 280 nm, hydroxycinnamic acids at 320 nm, flavonols at 360 nm, and anthocyanins at 520 nm. Photodiode detector spectra were measured over the wavelength range 200–600 nm. Phenolic compounds were identified using their retention time, UV-Vis characteristic and MS and MS^2^ properties, using data gathered in-house and from literature, as described in the previous work [29]. The identified compounds were quantified against a standard curve of peak areas of standard substances ((+)-catechin, (−)-epicatechin, procyanidin B1, B2 and C1, chlorogenic acid, cryptochlorogenic acid, neochlorogenic acid, cyanidin-3-glucoside, cyanidin-3-sambubioside, cyanidin-3-rutinoside, quercetin 3-rutinoside and quercetin 3-glucoside). The results were calculated as mg of compound in 1 g of freeze-dried fresh juice or purified juice. For biological studies, tested samples were dissolved in a PBS/DMSO (1:1 *v/v*) at concentrations presented in the descriptions of the tests carried out.

### 4.3. Cell Culture

Studies were performed with Saos-2 cell line—human osteosarcoma cells obtained from American Type Culture Collection (Manassas, VA, USA). Saos-2 cells were grown in low glucose Dulbecco′s Modified Eagle′s Medium (DMEM) with 10% fetal bovine serum (FBS) supplemented with 100 U/mL penicillin, 100 μg/mL streptomycin and 25 μg/mL amphotericin B. For osteogenesis stimulation medium, consisting of DMEM-low glucose completed with 2-Phospho-L-ascorbic acid (100 μM), L-proline (34.8 μM) and *β*2-glycerol phosphate (5 mM) was added [33]. For all experiments, if not stated otherwise, Saos-2 cells were seeded into a 96-well plate at a density of 10^4^ cells/well in complete medium and grown overnight. After 24 h, medium was changed with fresh osteogenic medium containing tested compounds for 8 days. The medium was changed every two days and fresh potion of compounds was added. Cells were maintained at 37 °C in a humidified incubator containing 5% CO_2_. All the experimental measurements were performed using the Synergy 2 BioTek Microplate Reader (BioTek, Winooski, VT, USA). Microscopic observations were performed using a contrast-phase and fluorescent microscope, Nikon TS100 Eclipse (Nikon, Tokyo, Japan) under ×200 magnification, if not stated otherwise.

### 4.4. Cell Viability and Proliferation

Saos-2 cells were seeded into a 96-well plate at a density of 10^4^ cells/well in complete medium and grown overnight. Then, the medium was changed into osteogenic medium and cells were incubated in the presence of *V. opulus* samples diluted in culture medium for 48 h. PrestoBlue (Life Technologies, Carlsbad, CA, USA) reagent was used for 30 min to quanitify the metabolic activity of cells according to the manufacturer’s instructions (fluorescence measurement at F530/590 nm). Cell proliferation was evaluated with the CyQUANT Direct Cell Proliferation Assay (Life Technologies, Carlsbad, CA, USA), according to the manufacturer’s instructions, by measuring the fluorescent signal at F485/528 nm.

### 4.5. Detection of Intracellular Reactive Oxygen Species Generation 

The effect of samples on the intracellular generation of reactive oxygen species (ROS) was checked with dichloro-dihydro-fluorescein diacetate (DCFH-DA) dye. After cell treatment with *V. opulus*, the medium was changed into phosphate buffer saline (PBS), 10 μM probe was added for 30 min and fluorescence at F485/530 nm was measured.

### 4.6. DNA Damage and Repair

*V. opulus* samples were investigated in terms of their ability to induce DNA repair in Sao-2 cells that had been exposed to mutagen–methylnitronitrosoguanidine (MNNG). The research was conducted according to Nowak et al. (2015) with some modifications [75]. So, as to examine DNA repair, the cells were damaged with 6.8 μM MNNG for 10 min on ice. Next, the cells were centrifuged (182× *g*, 4 °C, 15 min), resuspended in fresh medium and exposed (for 60 and 120 min at 37 °C) to the final concentrations without cyto- and genotoxicity. At the start (0 min), and, after 60- and 120-min incubations, aliquots of the suspensions were taken and, to stop the DNA repair in cells, the samples were placed in an ice bath. At each time interval, an alkaline comet assay (pH > 13) was performed and DNA repair was quantified by the determination of the extent of residual DNA damage. The positive control were cells exposed to MNNG, while the negative control consisted of Saos-2 cells in medium. In the comet assay, the final concentration of cells in each sample was adjusted to 10^5^ cells/mL. The comet assay was performed as previously described [75]. After each incubation, aliquots of suspended cells were centrifuged (182× *g*, 15 min, 4 °C), decanted, suspended in 0.75% LMP agarose and distributed onto slides precoated with 0.5% NMP agarose and immersed in lysing solution containing 2.5 M NaCl, 1% Triton X-100, 100 mM EDTA and 10 mM Tris, pH 10 (4 °C, 1 h). After lysis, the slides were subjected to horizontal gel electrophoresis and DNA was allowed to unwind for 20 min in an electrophoretic solution containing 300 mM NaOH and 1 mM EDTA. Electrophoresis was conducted at 4 °C for 30 min at an electric field strength of 0.73 V/cm (300 mA). Then, the slides were neutralized with distilled water for 5 min, stained with 1 mg/mL 4’,6’-diamidino-2-phenylindole (DAPI) and covered with cover slips. The comets were visualized at ×200 magnification with a fluorescence microscope (Nikon Eclipse Ci H600L, Nikon, Tokyo, Japan) attached to a digital camera (Nikon Digital Sight DS-U3, Nikon, Tokyo, Japan) and connected to a personal computer-based image analysis system, Lucia-Comet v. 7.0 (Laboratory Imaging, Prague, Czech Republic). Fifty images were randomly selected from each sample and the percentage of DNA in the comet tail was measured. The results were presented as mean ± standard error of the mean (S.E.M.).

### 4.7. Alizarin Red Cells Staining

After 8 days of incubation with *V. opulus* samples, the Saos-2 cells were washed with PBS and fixed with 5% formaldehyde solution for 30 min at room temperature [22,76]. After cell rinsing with water-staining, a solution of 1% alizarin red S in 2% ethanol (pH 4.0) was added for 30 min. Then cells were rinsed five times with destilled water and observed under a microscope. To quantify the matrix mineralization, cells were incubated with 100mM cetylpyridinium chloride for 1 h with gentle shaking. The absorbance of solubilized calcium-bound alizarin red S was measured at 570 nm. 

### 4.8. Estimation of Alkaline Phosphatase Activity

After treatment with *V. opulus* samples, Saos-2 cells were washed with PBS, then 1.0 mg/mL p-nitrophenyl phosphate (pNPP) in 0.2 M Tris buffer as the substrate for alkaline phosphatase (ALP) was added for 15 min and absorbance at 405 nm was measured [22]. To visualize ALP activity in cells, BCIP (5-bromo-4-chloro-3-indolyl phosphate)/ NBT (nitroblue tetrazolium) substrate was added.

### 4.9. Gene Expresssion Analysis

To study the influence of *V. opulus* on gene expression, Saos-2 cells were seeded into a 6-well plate at a density of 2 × 10^5^ cells/well in complete medium and grown overnight. After 24 h, medium was changed with fresh osteogenic medium containing tested compounds for 8 days. Total RNA was extracted from Saos-2 cells after 8 days incubation with *V. opulus* samples using GeneMatrix Universal RNA Purification Kit (Eurex Ltd., Gdansk, Poland), according to the manufacturer’s procedure. RNA samples were purified with Amplification Grade DNase I and reverse transcribed with NG dART RT Kit (Eurex Ltd., Gdansk, Poland). Real time RT-PCR was carried out using SG qPCR Master Mix (Eurex Ltd., Gdansk, Poland) on a BioRad CFX96 qPCR System (Bio-Rad, Hercules, CA, USA). Complementary DNA, representing 6 ng total RNA per sample, was subjected to 25–40 cycles of PCR amplification. Samples were first incubated at 95 °C for 40 s, then at 55 °C for 30 s, and finally at 72 °C for 30 s. To exclude non-specific products and primer-dimers, after the cycling protocol, a melting curve analysis was performed by maintaining the temperature at 52 °C for 2 s, followed by a gradual temperature increase to 95 °C. The threshold cycle (Ct) values for that gene did not change in independently performed experiments. The level of target gene expression level was calculated as 2^−ΔΔ*C*t^, where

ΔΔ*C*t = [*C*t(target) − *C*t(*GAPDH*)]_sample_ − [*C*t(target) − *C*t(*GAPDH*)]_control_ (1)

Gene expression was normalized using constitutively expressed glyceraldehyde-3-phosphate dehydrogenase (*GAPDH*) as a reference gene. The following primer sequences were used to determine the genes’ expression: *RUNX2* 5’-CAGTTCCCAAGCATTTCATCC-3’ (F) and 5’-TCAATATGGTCGCCAAACAG-3’ (R); *ALP* 5’-ACCTCGTTGACACCTGGAAG-3’ (F) and 5’-CCACCATCTCGGAGAGTGAC-3’ (R); *COL1A1 5’*-GCCAAGACGAAGACATCCCA-3’ (F) and 5’-CACCATCATTTCCACGAGCA-3’ (R); *RANKL* 5’-GAGTTGGCCGCAGACAAGA-3’ (F) and 5’-TTGGAGATCTTGGCCCAACC-3’ (R); *OPG* 5’-CAGCGGCACATTGGAC-3’ (F) and 5’-CCCGGTAAGCTTTCCATCAA-3’ (R); *Il6 5’–*TGGCTGAAAAAGATGGATGCT-3’ (F) and 5’-AACTCCAAAAGACCAGTGATGATT-3’ (R); *TNFα* 5’-CCCAGGCAGTCAGATCATCTTC-3’ (F) and 5’-AGCTGCCCCTCAGCTTGA-3’ (R); *OSTEONECTIN* 5’-GTGCAGAGGAAACCGAAGAG-3’ (F) and 5’-CGATAGGCCTCCTGAAAGC-3’ (R); *GAPDH* 5’-CCACCCATGGCAAATTCCATGGCA-3’ (F) and 5’-TCTAGACGGCAGGTCAGGTCCACC-3’ (R). Data analyses were obtained from at least three independent experiments.

### 4.10. Determination of Selected Proteins Levels

To study *V. opulus* influence on cytokine secretion, Saos-2 cells were seeded into a 24-well plate at a density of 4 × 10^4^ cells/well in complete medium and grown overnight. After 24 h, medium was changed with fresh osteogenic medium containing tested compounds for 8 days. On the last day of cell treatment, the medium was collected and protein concentrations of Il6 (Human IL6 ELISA kit, Biorbyt Ltd., Cambridge, GB) and TNFα (Human TNF alpha ELISA kit, Biorbyt Ltd., Cambridge, GB) were determined using ELISA kits, following the manufacturer’s instructions. Briefly, the collected medium samples were incubated for 2 h at room temperature to allow the cytokine binding to the well by the immobilized antibody. After washing, the plates were incubated for 1 h with biotinylated anti-human TNF alpha or anti-human Il6 antibodies, respectively. After washing, HRP-conjugated streptavidin or avidin–biotin–peroxidase complex was added, respectively, for 45 min. After washing, a 3′,5,5′-tetramethylbenzidine (TMB) substrate solution was added for blue color development. After the addition of the stop solution, the absorbance was measured at 450 nm. In cell lysates obtained with 0.1% Triton X-100 with PBS, the protein level was quantified with the Bradford assay. The cytokine levels were normalized to protein content.

### 4.11. Antimicrobial Activity of V. opulus Juice

In the research, twelve strains of pathogens were used. Within the pathogens there were seven Gram-negative: *Escherichia coli* ATCC 10536; *E. coli* ATCC 8739; *Pseudomonas aeruginosa* ATCC 15442; *P. aeruginosa* ATCC 24755; *Enterobacter cloacae* ATCC 13047; *Salmonella* Typhimurium ATCC 14028; *Salmonella* Enteritidis ATCC 13076. There were four Gram-positive species: *Staphylococcus aureus* ATTC 25923; *S. aureus* ATTC 6538; *Enterococcus faecalis* ATCC 29212; *Listeria monocytogenes* ATCC 19115. Additionally, one yeast strain—*Candida albicans* ATCC 10231—was engaged. The abovementioned strains were obtained from American Type Culture Collection (ATCC; Manassas, VA, USA). Moreover, six strains of lactic acid bacteria (LAB, Gram-positive) were applied: *Lactobacillus rhamnosus* GG (commercial strain); *Lactobacillus plantarum* ŁOCK 0981; *Lactobacillus brevis* ŁOCK 0983; *Lactobacillus paracasei* ŁOCK 0985; *Lactobacillus delbrueckii* ŁOCK 0987; *Lb. plantarum* ŁOCK 0989. *Lb. rhamnosus* GG was isolated from Dicoflor. The remaining strains were acquired from the collection of the Institute of Fermentation Technology and Microbiology (ŁOCK 105), Lodz University of Technology, Poland. Selected strains were stored with the usage of Cryobanks™ (Copan Diagnostics Inc., Murrieta, CA, USA) at −22 °C. Before analysis, both LAB as well as pathogens were activated and passaged twice in de Man, Rogosa and Sharpe broth (MRS; Merck–Millipore, Darmstadt, Germany), nutrient broth with glucose (Merck–Millipore, Darmstadt, Germany) or YPG (yeast extract–peptone glucose medium; yeast extract 10 g/L; peptone 20 g/L; glucose 20 g/L; pH 7.2), respectively for 24 h in 37 °C.

The antagonistic activity was investigated according to the agar well diffusion method. *Lactobacillus* sp. (10^6^ cells/mL) strains were spread over the entire surface of MRS agar in Petri dishes. After 15 min, 5 mm diameter holes were cut from the MRS medium with a cork borer and a volume of 100 µL of *Viburnum opulus* FJ and PJ samples (at concentration of phenolic compounds equal to 11 mg/mL) were introduced into the well in triplicate. The cultures were incubated at 37 °C for 24 h. The same procedure was applied to pathogens with the appropriate abovementioned agar media. Following the incubation, the diameter of the *V. opulus* sample on the growth inhibition zone was measured, the hole diameter was subtracted and the results were given in mm.

### 4.12. VEGF secretion 

Human umbilical endothelial cells (HUVEC) were grown in RPMI medium supplemented with 20% fetal bovine serum (FBS), 150 µg/mL endothelial growth factor, 100 U/mL penicillin, 100 µg/mL streptomycin and 25 µg/mL amphotericin B. For the VEGF secretion experiments, the cells were plated in 96-well plates at 5 × 10^3^ cells per well and cultured for 24 h in standard culture medium. The medium was changed to starving conditions for an additional 24 h and *V. opulus* samples were added for another 48 h. Culture supernatants were collected and VEGF levels were determined by the VEGF Human ELISA Kit (Life Technologies, Carlsbad, CA, USA), according to the manufacturer’s instructions.

### 4.13. Statistical Analysis

All the biological results are presented as means ± SEM, *n* ≥ 4. All calculations were evaluated for significance using one-way ANOVA, followed by Dunnett’s test with GraphPad Prism 6.0 software (GraphPad Software, Inc., La Jolla, CA, USA) at the significance level of * *p* ≤ 0.05, ** *p* ≤ 0.01, *** *p* ≤ 0.001. 

## 5. Conclusions

**Figure 11:**
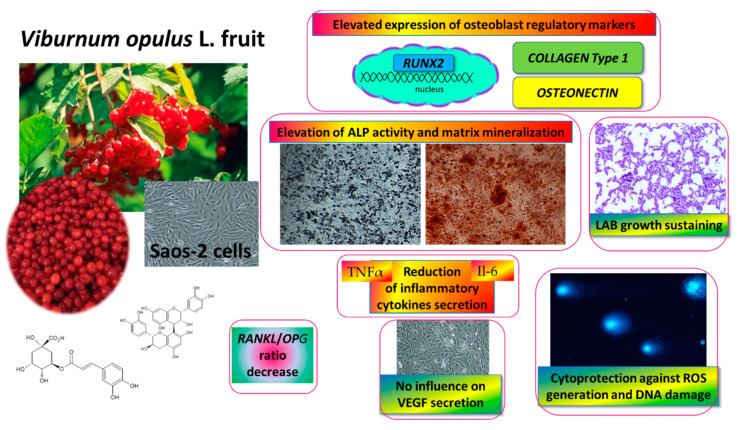
*V. opulus* fruit juice phenolic compounds as regulators of Saos-2 cell activity-proposed mechanism of action. *V. opulus* upregulates the mineralization of Saos-2 cells with an increase in the expression of the *RUNX2* transcription factor; diminishes the release of TNFα and Il6 inflammatory cytokines; possesses cytoprotective activity against ROS generation and DNA damage; sustains lactic acid beneficial microorganism growth.

This is the first study demonstrating the influence of *V. opulus* juice on osteosarcoma Saos-2 cell activity, as shown in Figure 11. As it was observed, fresh juice and purified juice were able to stimulate the mineralization of Saos-2 cells. Whereas the PJ preparation contained almost 90-times more phenolic compounds than fresh juice, its effective concentration was only 2.5-times lower than fresh juice. Because the composition of FJ and PJ is very complex (more than 30 phenolic compounds were identified) it would be premature to infer that the observed osteogenic potential reported here results only from the main components present in preparations, such as chlorogenic acid, procyanidins or catechins. Moreover, the detected *V. opulus* fresh juice biological impact might be related to the presence of other non-phenolic compounds, such as sugars, proteins, organic acids, minerals, or other phenolics, which were lost during solid-phase extraction and not detected in PJ. In this regard, potential synergetic activities and chemical interactions may be responsible for the observed cellular effect.

Both *V. opulus* fruit samples stimulated Saos-2 mineralization via the regulation of the expression of the major osteogenic factor, *RUNX2*. The main osteogenic differentiation markers, such as *ALP*, collagen type 1 and osteonectin, were elevated at transcription level. The observed decrease in the *RANKL*/*OPG* ratio may suggest the anti-resorptive ability of *V. opulus* fruits. Furthermore, the secretion of pro-inflammatory cytokines Il6 and TNFα, as well as intracellular oxidative stress, were diminished after cell incubation with samples. These observations reflect the potential of *V. opulus* fruit phenolics to decrease bone tissue demineralization, which may be induced by association with obesity inflammation and elevated ROS generation. What is more, *V. opulus* juice revealed protective properties against cellular damage by the stimulation of DNA repair. Because the release of VEGF protein by HUVEC cells was not affected by the samples, thus we can conclude that no disturbance of the angiogenesis process improves bone tissue formation or regeneration. The ability of *V. opulus* fruit juice phenolic compounds to inhibit pathogenic bacteria without the inhibition of lactic acid-beneficial microorganisms was also demonstrated. 

Taken together, our results contribute to elucidate the fact that *V. opulus* fruit juice’s molecular mechanism in Saos-2 cell mineralization process suggests its usage as a diet component potentiating bone health. Still, there is further need to check its osteogenic potential after in vitro digestion or incubation with gut microflora, as well as during in vivo studies with animal models with osteoporosis and induced obesity.

## Figures and Tables

**Table 1:** Phenolic compound contents in fresh juice and purified juice of *Viburnum opulus* fruit.

Phenolic Compound		Content (mg/g)	
		Fresh Juice (FJ)	Purified Juice (PJ)
**FLAVANOLS**	(+)-Catechin	0.657 ± 0.006	40.729 ± 0.596
	(−)-Epicatechin	0.135 ± 0.002	8.002 ± 0.116
	(Epi)catechin derivative I^a^	0.103 ± 0.001	6.998 ± 0.221
	(Epi)catechin derivative II^a^	0.080 ± 0.001	6.006 ± 0.165
	Gallocatechin gallate^a^	0.031 ± 0.000	1.876 ± 0.085
	Procyanidin dimer B1	0.759 ± 0.003	47.596 ± 0.148
	Procyanidin dimer B2	0.199 ± 0.002	11.540 ± 0.148
	Procyanidin dimer^b^	0.024 ± 0.001	1.602 ± 0.258
	B-type procyanidin dimer derivative I^b^	0.016 ± 0.000	2.071 ± 0.097
	B-type procyanidin dimer derivative II^b^	0.035 ± 0.000	2.293 ± 0.094
	Procyanidin trimer C1	0.033 ± 0.001	3.212 ± 0.351
	Procyanidin trimer I^c^	0.112 ± 0.001	6.866 ± 0.342
	Procyanidin trimer II^c^	0.030 ± 0.006	2.634 ± 0.270
	Procyanidin trimer III^c^	0.032 ± 0.000	1.796 ± 0.053
**HYDROXYCINNAMIC ACIDS**	Chlorogenic acid	8.039 ± 0.145	645.492 ± 1.984
	Cryptochlorogenic acid	0.004 ± 0.000	0.484 ± 0.023
	Neochlorogenic acid	0.007 ± 0.001	0,215 ± 0.019
	Caffeoylquinic acid^d^	0.745 ± 0.001	44.344 ± 0.176
	Caffeoylquinic acid derivative I^d^	0.015 ± 0.000	1.289 ± 0.058
	Caffeoylquinic acid derivative II^d^	0.024 ± 0.002	1.051 ± 0.008
	Caffeoylquinic acid derivative III^d^	0.017 ± 0.001	1.220 ± 0.020
	Caffeoylquinic acid derivative IV^d^	0.034 ± 0.000	3.306 ± 0.014
	Caffeoylquinic acid derivative V^d^	0.034 ± 0.000	3.268 ± 0.010
	Feruloylquinic acid I^d^	n.d.	5.722 ± 0.021
	Feruloylquinic acid II^d^	n.d.	0.528 ± 0.005
**FLAVONOLS**	Quercetin-3-vicianoside^e^	0.020 ± 0.000	1.266 ± 0.007
	Quercetin-3-galactoside^e^	n.d.	0.149 ± 0.011
	Quercetin-3-rutinoside	0.016 ± 0.000	0.921 ± 0.007
	Quercetin-3—rhamnoside^e^	0.007 ± 0.000	0.491 ± 0.002
**ANTHO-CYANINS**	Cyanidin-3-sambubioside	0.093 ± 0.000	7.010 ± 0.003
	Cyanidin-3-glucoside	0.139 ± 0.000	13.583 ± 0.799
	Cyanidin-3-rutinoside	0.068 ± 0.001	5.246 ± 0.016

n.d.—not detected; results are expressed as a mean ± standard deviation (*n* = 3). Content expressed as: *^a^*—equivalents of (+)-catechin; *^b^*—equivalents of procyanidin B1; *^c^*—equivalents of procyanidin C1; *^d^*—equivalents of chlorogenic acid; ^e^—equivalents of quercetin 3-glucoside.

**Table 2:** The antimicrobial activity of *V. opulus* FJ and PJ.

Strain	Inhibition Zone [mm]	
	FJ	PJ
*S. aureus* ATTC 25923	2.0	7.0
*S. aureus* ATTC 6538	12.3	9.0
*L. monocytogenes* ATCC 19115	0	8.7
*Ent. faecalis* ATCC 29212	6.0	10.0
*E. coli* ATCC 10536	0	0
*E. coli* ATCC 8739	0	0
*P. aeruginosa* ATCC 15442	0	0
*P. aeruginosa* ATCC 24755	0	0
*E. cloacae* ATCC 13047	0	0
*S. typhimurium* ATCC 14028	0	0
*S. enteritidis* ATCC 13076	0	0
*C. albicans* ATCC 10231	0	0
*Lb. rhamnosus* GG	0	0
*Lb. plantarum* ŁOCK 0981	0	0
*Lb. brevis* ŁOCK 0983	0	0
*Lb. paracasei* ŁOCK 0985	0	0
*Lb. delbrueckii* ŁOCK 0987	0	0
*Lb. plantarum* ŁOCK 0989	0	0

## Abbreviations

ALP	alkaline phosphatase
FJ	fresh juice
Il6	interleukin 6
NF-κB	nuclear factor kappa B
OPG	osteoprotegerin
RANKL	receptor activator of nuclear factor kappa-Β ligand
RUNX2	Runt-related transcription factor 2
ROS	reactive oxygen species
TNFα	tumor necrosis factor α
VEGF	vascular endothelial growth factor
PJ	purified juice
